# An information and communication technology-based centralized clinical trial to determine the efficacy and safety of insulin dose adjustment education based on a smartphone personal health record application: a randomized controlled trial

**DOI:** 10.1186/s12911-017-0507-4

**Published:** 2017-07-18

**Authors:** Gyuri Kim, Ji Cheol Bae, Byoung Kee Yi, Kyu Yeon Hur, Dong Kyung Chang, Moon-Kyu Lee, Jae Hyeon Kim, Sang-Man Jin

**Affiliations:** 1 Division of Endocrinology and Metabolism, Department of Medicine, Samsung Medical Center, Sungkyunkwan University School of Medicine, Seoul, 135-710 Republic of Korea; 2 Division of Endocrinology and Metabolism, Department of Medicine, Samsung Changwon Hospital, Sungkyunkwan University School of Medicine, Changwon, Republic of Korea; 3 Department of Digital Health, SAIHST, Sungkyunkwan University, Seoul, Republic of Korea; 4 Division of Gastroenterology, Department of Medicine, Samsung Medical Center, Sungkyunkwan University School of Medicine, Seoul, Republic of Korea; 5 Department of Clinical Research Design & Evaluation, SAIHST, Sungkyunkwan University, Seoul, Republic of Korea

**Keywords:** Diabetes mellitus, eCRF, ICT-based clinical trial, Insulin, Mobile applications, Personal health record, Hypoglycemia

## Abstract

**Background:**

A Personal Health Record (PHR) is an online application that allows patients to access, manage, and share their health data. PHRs not only enhance shared decision making with healthcare providers, but also enable remote monitoring and at-home-collection of detailed data. The benefits of PHRs can be maximized in insulin dose adjustment for patients starting or intensifying insulin regimens, as frequent self-monitoring of glucose, self-adjustment of insulin dose, and precise at-home data collection during the visit-to-visit period are important for glycemic control. The aim of this study is to examine the efficacy and safety of insulin dose adjustment based on a smartphone PHR application in patients with diabetes mellitus (DM) and to confirm the validity and stability of an information and communication technology (ICT)-based centralized clinical trial monitoring system.

**Methods:**

This is a 24-week, open-label, randomized, multi-center trial. There are three follow-up measures: baseline, post-intervention at week 12, and at week 24. Subjects diagnosed with type 1 DM, type 2 DM, and/or post-transplant DM who initiate basal insulin or intensify their insulin regimen to a basal-bolus regimen are included. After education on insulin dose titration and prevention for hypoglycemia and a 1-week acclimation period, subjects are randomized in a 1:1 ratio to either an ICT-based intervention group or a conventional intervention group. Subjects in the conventional intervention group will save and send their health information to the server via a PHR application, whereas those in ICT-based intervention group will receive additional algorithm-based feedback messages. The health information includes level of blood glucose, insulin dose, details on hypoglycemia, food diary, and step count. The primary outcome will be the proportion of patients who reach an optimal insulin dose within 12 weeks of study enrollment, without severe hypoglycemia or unscheduled clinic visits.

**Discussion:**

This clinical trial will reveal whether insulin dose adjustment based on a smartphone PHR application can facilitate the optimization of insulin doses in patients with DM. In addition, the process evaluation will provide information about the validity and stability of the ICT-based centralized clinical trial monitoring system in this research field.

**Trial registration:**

Clinicaltrials.gov NCT 03112343. Registered on 12 April 2017.

**Electronic supplementary material:**

The online version of this article (doi:10.1186/s12911-017-0507-4) contains supplementary material, which is available to authorized users.

## Background

Despite the development of various diabetes medications, insulin therapy is still the only effective treatment not only in patients with type 1 diabetes mellitus (T1DM), but also in patients with advanced type 2 diabetes mellitus (T2DM) that is inadequately controlled by oral glucose-lowering agents due to disease progression [1–3]. However, due to the failure of insulin dose titration and fear of hypoglycemia, insulin treatment is perceived as a complex and reluctant therapy by both patients and doctors [4, 5]. To overcome these barriers, frequent and intensive self-monitoring of glucose and detailed feedback on insulin dosage are critical [6]. Therefore, there is a strong clinical need for a technological approach to enhance the timely communication between patients and their healthcare providers about insulin therapy. Several studies have reported the efficacy of telephone counseling or text messaging based on paper-based collection of self-monitored glucose data [7], rather than automatic transmission of glucose data [8]. In contrast, there have been several randomized controlled trials of bolus-calculator applications in patients with T1DM [9]. However, most of these studies used applications without automatic data uploading functionality [10], and there have been continuous concerns regarding the safety of bolus calculator applications [9]. Until the safety of automated insulin dose calculator applications is sufficiently documented, there is a clinical need for mobile applications to enhance patient education around insulin dose adjustment.

A personal health record (PHR) is an online application that allows patients to access, manage, and share their own health data [11]. Unlike in self-care medical applications, focusing on incomplete self-care data, providing little useful information to clinicians, PHR not only enhances shared decision making with healthcare providers in actual clinical practice, but also enables remote monitoring and at-home-collection of detailed data in clinical trials [12]. These benefits of PHRs in both clinical practice and trials can be maximized in the education of insulin dose adjustment for patients with DM who are starting or intensifying their insulin regimen, as frequent self-monitoring of glucose level, self-adjustment of insulin dose, and at-home data collection with minimized visits to the hospital are important for description of details on glycemic control, including hypoglycemia, diet, and activity. However, the role of PHR in patients starting or intensifying insulin therapy has received less attention than in patients with early-stage T2DM, despite the strong clinical need for PHR in patients with T1DM or insulin-treated T2DM.

The aim of this study is to examine the efficacy and safety of insulin dose adjustment education based on a smartphone PHR in patients with DM who initiate or intensify their basal insulin regimen and to confirm the validity and stability of the information and communication technology (ICT)-based centralized clinical trial monitoring system by analyzes using PHR- and questionnaire-based evaluation.

## Methods

### Hypotheses

Our primary hypothesis is that the ICT-based intervention group will produce a greater proportion of patients who reach optimal insulin dose within 12 weeks compared with the conventional intervention group. Our secondary hypothesis is that the ICT-based intervention group will have a greater proportion of patients who reach HbA1c <7% without severe hypoglycemia at weeks 12 and 24, improved glycometabolic control (including levels of fasting glucose, HbA1c, lipid parameters, body weight, lean body mass, fat mass, and blood pressure), step count, exchange units for each food category, and fewer hypoglycemic events or acute complications relative to the conventional intervention group.

### Study design

**Fig. 1:**
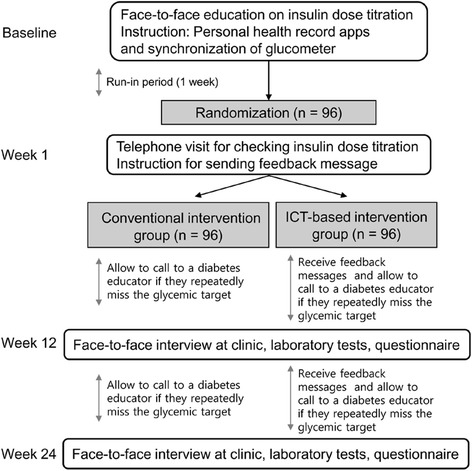
Information and communication technology-based centralized monitoring system. The CRC registers the subject in the integrated management system, registers personalized conditions such as target blood glucose, and logs in the personal health record app through the newly created user ID and password. The PHR app sends login information to the home monitoring system and returns the survey ID. The home monitoring system receives user information (personal information, screening number, etc.) from the integrated management system. The PHR app sends login information and survey ID to the hospital monitoring system and automatically registers the patient. The CRC registers personalized conditions, such as target blood glucose and subject classification, in the hospital monitoring system. Blood glucose values by glucometer, step count by Samsung Health app, insulin regimen, hypoglycemia diary, and food diary inputted by the patient are transmitted to the home and hospital monitoring systems through the HL7 v2 message. Based on the received blood glucose values, the hospital monitoring system sends an algorithm-based feedback message, and the PHR app shows the feedback message. The PHR app that receives the feedback message transmits the HL7 v2 message to the home monitoring system. All information and feedback messages saved in the home monitoring system are stored in the eCRF. ﻿*CRC* Clinical research coordinator, *PHR* Personal health record

This is a 24-week, open-label, randomized, multi-center ICT-based clinical trial conducted in two different university-affiliated hospitals, Samsung Medical Center and Samsung Changwon Hospital. There are three follow-up measures: baseline, post-intervention at week 12, and at week 24. Subjects diagnosed as T1DM, T2DM, and/or post-transplant DM who are initiating basal insulin or intensifying their insulin regimen to a basal-bolus regimen will be given education on insulin injection, dose adjustment, and prevention of hypoglycemia and will be provided a Bluetooth-enabled glucometer at visit 1 for screening. Subjects will receive instructions to measure daily glucose level using a home glucometer, to record insulin regimen and dose, complete the hypoglycemia diary in the application if blood glucose <70 mg/dL or a hypoglycemic event occurs, and to synchronize data so it automatically transfers to the system. Subjects who synchronized their information daily during a 1-week run-in period will be accepted into the clinical trial and randomly assigned to either the ICT-based intervention group or the conventional intervention group at a ratio of 1:1. After randomization during week 1, telephone counselling will be provided for re-instructing insulin dose adjustment and re-confirming participants’ use of the at-home measurement device and PHR applications (visit 2; televisit). Subjects in the ICT-based intervention group will receive algorithm-based feedback messages when their glucose levels are out of the target range, in addition to recording, saving, and sending their data to the server via PHR applications. Subjects in the conventional intervention group will only record, save, and send their data to the server via PHR applications, without feedback messages. Investigators will examine the saved health information, including level of blood glucose, insulin dose, details on hypoglycemia recorded in the hypoglycemia diary, food diary, and number of steps, transferred through the PHR applications. At each clinical visit, anthropometric parameters, current medication use including insulin dose and other glucose-lowering agents, vital signs, body composition, and a questionnaire assessing satisfaction with the investigation will be examined, and blood tests will be performed. Figure 1 shows an overview of the study.

### ICT-based centralized clinical trial monitoring system

**Fig. 2:**
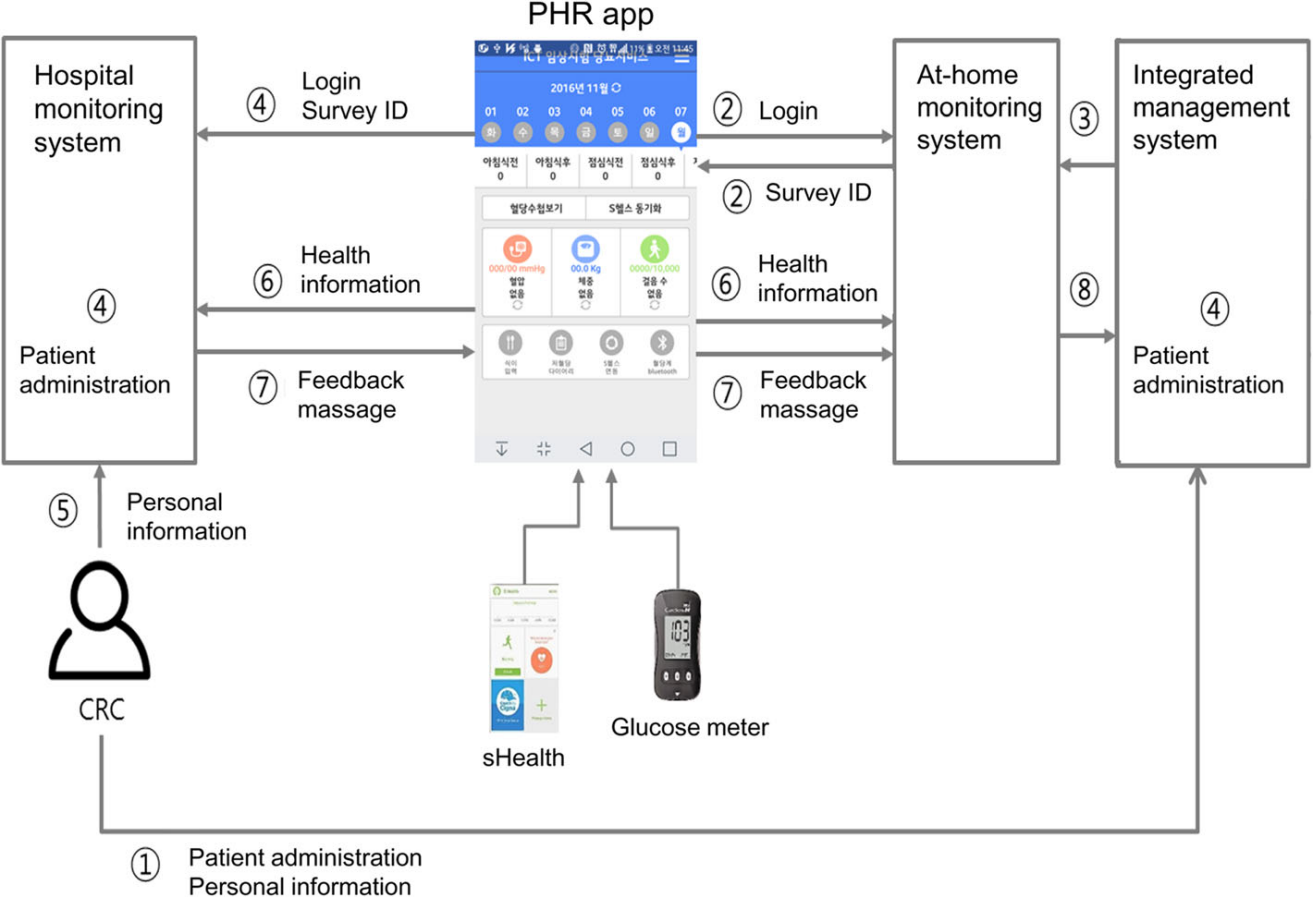
Study flow chart

A centralized clinical trial monitoring system based on ICT allows the investigators to store and retrieve personal medical information transmitted from a medical device at an out-of-hospital location (e.g., at home) and to obtain reliable clinical data. This system is expected to be useful for clinical decision making (Fig. 2). The ICT-based centralized clinical trial monitoring system for clinical trials used in this trial consists of 1) a PHR application that acts as a gateway to identify the subjects of the clinical trial and transmit biometric information to the server; 2) at-home measurement device (Bluetooth-enabled blood glucometer); 3) a home monitoring system server where transmitted data is stored, monitored, and transferred; 4) an electronic case report form (eCRF) system (EDC) that extracts and implements the data necessary for clinical trials from the home monitoring system data; and 5) an integrated management server, in which all the above data are collected and managed. The at-home measurement device required for this clinical trial is a blood glucometer linked with Bluetooth, which measures and transmits blood glucose levels to the PHR application with every measurement. Therefore, syncing to the PHR application with Bluetooth is required to save and transmit data to the server automatically. The step count taken per day acquired by the Samsung Health application (Samsung Electronics, Korea) is automatically linked to the PHR application. The Android-based ‘PHR app,’ having recently developed new functions making it capable of interoperability with a wide range of devices, transmission of PHR data to a clinical trial server, and a graphic interface for providing information to medical staff, will be applied for this clinical trial. In addition, subjects can record their insulin protocol, food diary, and ‘hypoglycemia diary’ in the PHR app. The home monitoring system accumulates transferred biometric data, including blood glucose levels measured from the at-home measurement device, step count, and recorded data, including insulin regimen, food diary, and hypoglycemia diary for each registered subject through an integrated management system and transmits the data to the eCRF system via HL7 v2 message, a standardized protocol. For the ICT-based intervention group, PHR app information is sent to the investigator web page in the home monitoring system, and the investigator can view the subject’s data. In addition, algorithm-based feedback messages are transmitted to investigators, as well as subjects in the ICT-based intervention group when blood glucose level is not within the target range. Push feedback messages generated by the home monitoring system server will be displayed on the screen of the subject’s PHR app. eCRF is necessary for inputting and storing clinical trial data for each visit per subject through the integrated management system. Only the investigator or designated person can access the eCRF system to input, store, and examine a subject’s data. The Clinical trial management system (CTMS) is a superordinate system for managing all data, from subject registration and randomization, for transmitting data to the home monitoring system and eCRF and for integrating and managing all data for each measurement per subject. In addition, the CTMS is responsible for the management of participating institutions and investigators, monitoring, and adverse reaction monitoring and reporting. For system data security, the PHR app, the investigator web page, the home monitoring system, the eCRF system, and the integrated CTMS all protect personal medical information and maintain high standards of confidentiality by system log recording methods through automatic logoff, control and authentication on access, data encryption, and data audits.

### Participants

#### Eligibility criteria

The inclusion criteria are as follows: 1) age between 18 and 69 years; 2) diagnosis of T1DM, T2DM, and/or post-transplant DM (post-transplant DM includes both individuals diagnosed with T1DM or T2DM before organ transplantation and those first diagnosed with DM after organ transplantation); 3) initiation or intensification of insulin regimens, including once-daily basal insulin (including initiation of basal insulin only or basal insulin plus rapid-acting insulin 1 to 3 times per day and intensification to multiple insulin injections from twice-daily premixed insulins or once-daily basal insulin; patients will be eligible unless the insulin regimen was initiated more than 1 month prior to study participation, with insulin dose adjustment education provided by registered diabetes educators); 4) most recent hemoglobin A1c (HbA1c) ≥8.0% based on the National Glycohemoglobin Standardization Program (NGSP) at least 3 months prior to participation (recent HbA1c values measured <3 months prior to screening are permitted); and 5) use of an Android-based smartphone manufactured by Samsung Electronics (Seoul, Republic of Korea; Galaxy S4 or later). Exclusion criteria include the following: 1) initiating twice-daily pre-mixed or intermediate-acting insulin therapy; 2) use of an insulin pump; 3) history of alcohol or drug abuse 1 year prior to participation; 4) history of psychological disorder (e.g., schizophrenia, depression, or bipolar disorder); 5) history of severe visual or hearing impairment; 6) history of active malignancy; 7) pregnancy; and 8) any condition, in the investigator’s opinion, not suitable for enrollment eligibility.

### Recruitment

Participants will be patients diagnosed with T1DM, T2DM, and/or post-transplant DM if they initiate basal insulin or intensify their insulin regimen to a basal-bolus regimen at the tertiary hospitals Samsung Medical Center or Samsung Changwon Hospital in Korea. Individuals who are willing to participate in the clinical trial and who meet the inclusion criteria will receive written information about the study and must provide informed consent, as approved by the ethics committee [Additional file 1].

### Outcome variables

The primary outcome will be the proportion of patients who reach the optimal insulin dose within 12 weeks of enrolling in the study, without severe hypoglycemia or unscheduled visits to the clinic. Achievement of the optimal insulin dose is defined as follows: A. For a patient who uses basal insulin only or basal insulin with pre-meal rapid-acting insulin once per day, 1) three or more fasting blood glucose values within the target range (80 to 130 mg/dL or 100 to 140 mg/dL) during week 12 (last 7 days prior to visit 3), 2) no nocturnal (from 11 pm to 7 am) or pre-breakfast hypoglycemia (<70 mg/dL) during week 12, and 3) variation of basal insulin dose (defined as the difference between the highest and lowest basal insulin doses) <10% of the mean total basal insulin dose during week 12. B. For a patient who uses multiple daily injections, including basal insulin with pre-meal rapid-acting insulin at least twice per day, the criteria are those in A plus 4) three or more days in which the correction dose of pre-meal insulin is required less than three times per day during week 12, and 5) no daytime (from 7 am to 11 pm) hypoglycemia (<70 mg/dL) during week 12.

The secondary outcome will be the proportion of patients who reach HbA1c <7% without severe hypoglycemia (unrecoverable hypoglycemia without the help of others) at weeks 12 and 24. Other secondary outcomes include mean fasting blood glucose values for three consecutive days prior to weeks 12 and 24; levels of HbA1c at weeks 12 and 24; the proportion of patients having hypoglycemia (total, asymptomatic, daytime, nighttime, severe hypoglycemia, and coma/convulsions) at weeks 12 and 24; the number of steps taken during weeks 1–12 and weeks 13–24; recorded exchange unit by food group (grain, fish, vegetable, fat, milk, and fruit groups) in each breakfast, lunch, and dinner on 3-day food diary at weeks 12 and 24; daily insulin dose, body weight, and blood pressure at weeks 12 and 24; lipid profiles including total cholesterol, high-density lipoprotein (HDL) cholesterol, triglycerides, and low-density lipoprotein (LDL) cholesterol at weeks 12 and 24; body composition, including lean body mass and fat mass, at weeks 12 and 24; satisfaction evaluation by Diabetes Treatment Satisfaction Questionnaire (DTSQ) [13–16] and a questionnaire for ICT-based centralized clinical trial monitoring at weeks 12 and 24; the number of self-monitoring blood glucose measurements; the mean duration of medical consultations by doctors at the outpatient clinic; and the number and mean duration of telephone counselling by diabetes educators at weeks 12 and 24.

### Sample size assumptions

In a previous study that utilized mobile phones for insulin titration intervention for subjects with type 2 diabetes, proportions of patients reaching optimal insulin dose within 12 weeks were 88% and 37% in the intervention and usual care arms, respectively [7]. In the present clinical trial, for both the intervention and control groups, we provide telephone counseling 1 week after patient education and allow telephone counseling if the blood glucose level does not meet the target level in both ICT-based intervention and conventional intervention groups. Therefore, the rate of reaching the primary endpoint of the control group is expected to be 55%, higher than that of the reference, and the estimated proportion of the intervention group that meets optimal insulin dose is 88%, which is similar to the reference. With these assumptions, a sample size of 38 subjects per group is needed with a two-sided alpha threshold of 0.05 with 90% power. We aimed to recruit 96 participants (48 per group) to allow for a dropout rate of 20% during follow-up (Samsung Medical Center, *n* = 76; Samsung Changwon Hospital, *n* = 20) [17].

### Data collection

### Demographic and medical survey

Demographic and medical information, including age, smoking and alcohol consumption, past and present medical history (e.g., hypertension, dyslipidemia), family history of diabetes, and diabetes medication including oral glucose-lowering agents and insulin, other concomitant medications within 6 months of registration for the trial, and any history of complications due to diabetes (macrovascular and microvascular complication) will be collected.

### Anthropometric and vital sign measurements

At visits 1, 3, and 4, height, body weight, blood pressure, and pulse will be measured, and a brief physical examination will be performed. Body composition examination will be performed by bioelectrical impedance analysis (Table 1).

**Table 1:**
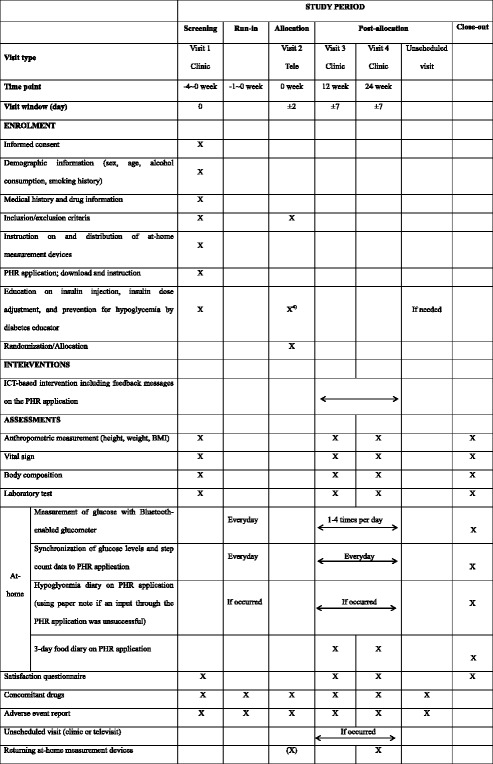
Study schedule

PHR: Personal health record

### Laboratory tests

At visits 1, 3, and 4, blood samples are collected after at least 8 h of fasting to determine fasting glucose, HbA1c, white blood cell count (WBC), hemoglobin, hematocrit, platelet count, total protein, albumin, globulin, blood urine nitrogen (BUN), creatinine, uric acid, total bilirubin, aspartate aminotransferase (AST), alanine transaminase (ALT), alkaline phosphatase (ALP), calcium, phosphate, total cholesterol, triglycerides, HDL cholesterol, and LDL cholesterol. At visit 1, a urine hCG test is performed as a pregnancy test in women with childbearing potential.

### Questionnaire for satisfaction with diabetes treatment and ICT-based centralized monitoring system

Subjects complete the DTSQ and the satisfaction questionnaire for the ICT-based centralized monitoring system to assess the impact of the intervention on the self-management of diabetes at visits 1, 3, and 4 [Additional file 2]. As the DTSQ has 2 versions, one assessing current status (DTSQs) and one assessing change (DTSQc), the DTSQs will be used at baseline (visit 1), visit 3, and endpoint (visit 4), and the DTSQc will be used at endpoint (visit 4).

### Education on insulin injection, dose titration, and prevention of hypoglycemia

At the screening visit (visit 1), all subjects are given face-to-face education on insulin injection, dose titration, and prevention of hypoglycemia. The initial daily basal insulin dose will be 10 units per day or 0.1–0.2 units per kg of body weight. The initial daily basal insulin dose can be increased when the fasting blood glucose level is 250 mg/dL or more at initiation, but it should not exceed 0.3 units per kg per day. The initial pre-prandial rapid-acting insulin dose will be 4 units or 0.1 unit per kg or 10% of the basal insulin dose. For patients who have already used insulin before enrollment, they will start on their previous insulin dose and adjust from there. Subjects in the study are instructed to titrate their insulin dose based on the glycemic target of fasting glucose 80–130 mg/dL in the morning, for those who are under 65 years of age and do not have a history of severe hypoglycemia that cannot be recovered without help. The target of fasting glucose level 100–140 mg/dL in the morning will be applied for subjects with 1) history of severe hypoglycemia; 2) age ≥ 65 years; 3) duration of diabetes mellitus over 15 years; 4) decreased renal function (estimated glomerular filtration rate < 45 mL/min/1.73 m^2^); 5) BMI <18.5 kg/m^2^; 6) unawareness of hypoglycemia; 7) unpredictable changes in blood glucose level; and 8) difficulties understanding the education provided.

### Distribution and use of at-home measurement device and personal health record apps

For all subjects, at-home measurement device including a Bluetooth-enabled glucometer will be provided and instruction including use of the device, synchronization, and automatic transmission of data to the server will be provided.

### Run-in period

Subjects must examine level of blood glucose through a Bluetooth-enabled glucometer for a 1-week run-in period and synchronize the measured glycemic information to the PHR app for automatic transmission to the server at least once a day during that week. If synchronization is unsuccessful, subjects should use manual input to transfer data beyond the pre-planned frequency; otherwise, they will be considered as a screening failure.

### Interventions

#### Randomization

Educators provide telephone counseling after the 1-week run-in period. Before the telephone counseling, investigators check the synchronization of the at-home measurement device and the PHR application through the web page for administrators. For those who are suitable for the clinical trial based on daily synchronization, block randomization will be performed. The subjects will be randomly allocated to an intervention with ICT-based centralized monitoring or not, at a ratio of 1:1, per the Interactive Web Response System (IWRS). The randomization is stratified according to the research institute (Samsung Changwon Hospital: Samsung Medical Center = 20:76) and the use of pre-prandial insulin two or more times per day.

### Telephone counseling and re-introduction of intervention

At week 1, educators provide telephone counseling to all subjects to assess concomitant medication use or any adverse events and to re-instruct insulin dose adjustment and PHR apps. Subjects who are screening failures must return the at-home measurement device to the clinic. All participants are instructed to call the diabetes educator if the measured blood glucose level is <70 mg/dL more than once, or if the blood glucose level is not in the range of the glycemic target more than twice. Also, unscheduled visits to the clinic are available if a subject has one or more severe incidents of hypoglycemia (requiring other help for recovery) during the study period, hypoglycemia (<70 mg/dL) two or more times per week, or experiences fasting blood glucose >200 mg/dL three or more times in the morning and wants to see a doctor during weeks 1–12. Unscheduled visits to the clinic are also allowed for subjects who have difficulty in insulin dose adjustment, despite two or more unscheduled tele-visits. All subjects will record a 3-day food diary regarding the exchange unit for each nutrient group (e.g., grain, fish, vegetable, fat, milk, and fruit groups) in the breakfast, lunch, and dinner prior to visits 3 and 4.

### ICT-based intervention group

Subjects allocated to the ICT-based intervention group will be informed about algorithm-based feedback messages, and that a diabetes educator can call the subject if the feedback message is not checked more than once. The detailed algorithms of the feedback messages are available in the supplementary information [Additional file 3]. Push messages generated by the home monitoring system server will be displayed on the screen of the subject’s PHR apps. If the synchronized blood glucose level is <70 mg/dL, the hypoglycemia diary is activated automatically and can also be activated by a participant if he or she experiences symptoms of hypoglycemia. Whether hypoglycemic symptoms (e.g., cold sweating, severe hunger, shivering, and panic) have occurred, whether they occur during the night or sleep, and whether subjects need help to recover from hypoglycemia can be recorded in the hypoglycemia diary from PHR apps. Moreover, additional telephone consultation after the feedback message ‘Unscheduled tele-visit’ will be performed and recorded on the ‘Unscheduled tele-visit’ page of eCRF when diabetes educators check the transferred data on the administrator webpage daily and find the data meet the following criteria: 1) there is no record of blood glucose and insulin dose within 2 days after the feedback message; 2) the blood glucose and insulin doses recorded for 2 days after the feedback message do not follow the pre-informed instructions; and 3) hypoglycemia occurs, but the hypoglycemia diary is not recorded.

### Data acquisition and evaluation

For all subjects, at each clinical visit, anthropometric parameters, current medication use (including types of insulin and insulin dose), hypoglycemic events, vital signs, body composition, questionnaire for satisfaction with the investigation, and any adverse events are examined, and blood tests are performed. The mean duration of medical consultations by a doctor at an outpatient clinic and the number and mean duration of telephone counselling calls by diabetes educators are also evaluated. Prior to the post-interventional clinical visit at weeks 12 and 24, a clinical research coordinator (CRC) will check whether subjects continuously run and synchronize their PHR apps, the number of synchronizations, transmitted data of insulin dose, parameters regarding hypoglycemia and the 3-day food diary, and the measured number of steps taken during weeks 1–12 and weeks 13–24 in both intervention groups. For the ICT-based intervention group, the CRC will identify the types of algorithm-based feedback messages, whether subjects act on the feedback, and the recorded data from the hypoglycemia diary.

### Evaluation of hypoglycemia

Hypoglycemia is defined using the following criteria: 1) asymptomatic hypoglycemia (if blood glucose is measured as <70 mg/dL without clinical symptoms); 2) symptomatic hypoglycemia (the symptoms of hypoglycemia (e.g., sweating, severe hunger, shivering, and panic) including blood glucose level < 70 mg/dL or not confirmed); 3) daytime hypoglycemia (7 am-11 pm; hypoglycemia before sleep after the wake-up); 4) nocturnal hypoglycemia (11 pm-7 am; hypoglycemia from sleep to wake-up); 5) severe hypoglycemia (help is needed to restore glucose level). If subjects have hypoglycemia meeting the above criteria, they use the hypoglycemia diary in the PHR app to record the details of the hypoglycemic event. At each clinic visit at weeks 12 and 24, the incidence and type of hypoglycemia are identified and recorded via face-to-face interview.

### Evaluation of the stability of ICT-based centralized clinical trial monitoring

At week 12 and 24 clinic visits, the CRC will identify the frequency of hypoglycemia, insulin dose, body weight, and blood pressure in the face-to-face interview; analyze the consistency of the data examined by the CRC and transmitted data from the PHR app, including the hypoglycemia diary; and verify the efficacy of the ICT-based centralized clinical trial monitoring. The satisfaction questionnaire about the DTSQ and ICT-based centralized clinical trial monitoring will be evaluated.

### Economic evaluation of ICT-based centralized clinical trial monitoring

Expenses associated with ICT-based centralized monitoring system and costs for additional admission, outpatient clinical examination, and transportation by insulin dose adjustment training based on PHR apps in both two groups will be evaluated. The same individuals will be analyzed twice using ICT-based and questionnaire/medical record-based evaluation to confirm the stability and validity of the ICT-based centralized clinical trial monitoring system.

### Discontinuation of subjects

Discontinuation of subjects comprises withdrawals as requested by the subject or by the investigator, loss to follow-up, and death. In addition, discontinuation of subjects also includes the cases in which the subject has experienced an adverse event that requires early termination because continued participation imposes an unacceptable risk to the subject’s health or the subject is unwilling to continue because of the adverse event. The reason for withdrawal or discontinuation of the subject from the study should be recorded in the eCRF. The investigator and site staff compile the case records, clinical trial progress, and results of the study performed until the time of discontinuation.

### Adverse event assessment and safety follow-up

At each televisit and clinical visit, concomitant medications and adverse events / severe adverse events will be inquired and assessed. The investigator or site staff will be responsible for detecting, documenting, and reporting on adverse events/ severe adverse events using the respective adverse event page in the eCRF. Investigators must report all new severe adverse events or pregnancies for which they have been notified at the safety follow-up.

### Data monitoring

The clinical research assistant (CRA) will conduct data monitoring to ensure that the human rights and safety of the study subject are being protected, the clinical trial is conducted in accordance with the approved clinical trial protocol and applicable regulatory requirements, and data are accurate, authentic, and complete, independent from competing interests. Monitoring should be carried out when it is necessary to check the CRF created at the time of each patient registration and to check the progress of the study. Periodic monitoring is performed after the first patient is registered for each institution to ensure the quality and reliability of clinical trial results.

### Audit

Any aspect of the clinical trial may be subject to audits to assure compliance with the KGCP and related regulations regarding the collection, recording, documentation and reporting of clinical trial data. The investigators and the site staffs should cooperate with the activities of CRA and give internal and external auditors direct access to all source documents at the trial site to examine, analyze, verify any records and reports relevant to the clinical trial. According to the results of the investigation, the investigator and site staff have an obligation to supplement and correct the clinical trial-related documents and immediately take the appropriate action. Any records and reports from the clinical trial must be retained at research institute for at least 10 years after completion of the study.

### Data analysis

All continuous variables will be presented as mean ± standard deviation, and categorical variables will be presented as frequency and proportion. For analyzing primary outcomes, the percentage of patients who reached the optimal insulin dose within 12 weeks is presented, and the difference between the two groups is compared using Pearson’s chi-square test or Fisher’s exact test if the frequency is low (<5). Differences are analyzed using Pearson’s chi-square test or Fisher’s exact test for categorical variables and Student’s t-test or Wilcoxon’s rank-sum test for continuous variables of secondary outcome variables. Comparisons between the two groups will be performed using a stratification analysis based on the use of pre-prandial insulin two or more times per day and each research institute. Prespecified subgroup analyses, including use of immunosuppressants (including steroids); type of DM; HbA1c >9%; use of pre-prandial insulin; aged over 65, 60, or 55; type of basal insulin; and stage of chronic kidney disease at baseline are performed. For handling missing data, last-observation-carried-forward (LOCF) imputation method would be performed.

### Ancillary and post-trial care

The investigator should ensure that subjects who are dropped out of clinical trials receive other appropriate care. Subjects who have completed clinical trials will receive appropriate care if treatment continues to be required by the investigator’s judgement.

## Discussion

In this study, the benefit of PHR will be investigated in patients with T1DM or insulin-treated T2DM initiating or intensifying insulin therapy, in which strict blood control with an avoidance of hypoglycemia and life style modification are crucial for the prevention of progressive diabetes-related complications and its enormous burden on the public health care system [18–20]. In addition, PHR not only allows for shared decision making on insulin dose adjustment, but also allows for detailed remote data collection, including blood glucose level, daily insulin dose, and life logs with minimized human effort to collect data via face-to-face interviews and/or paper-based records [11, 21]. In this study, PHR will also be used in a conventional intervention group for data collection, with the automated feedback feature disabled. This study thus will provide a valuable opportunity to evaluate the stability of PHR-based collection of life log variables and to establish the role of PHR in ICT-based centralized clinical trials based on remote patient monitoring.

With a large database of PHR from patients, effective access, handling, and storage of the data by an ICT-based centralized monitoring system are needed to approve its efficacy and safety. In the previous study regarding PHR among subjects starting basal insulin, patients used dedicated tablet computers for viewing their glucose patterns; however, the tablets were inconvenient to carry and often experienced difficulties connecting with the server [22]. In the previous study, the control group was not allocated to use PHR, so a direct comparison of the PHR-based intervention with the conventional intervention was not possible, as the method of data collection was not identical between the groups [22]. HbA1c and DTSQ evaluation were the outcome variables of the study, similar to our trial, but the PHR-based collection of hypoglycemia data, which is important for insulin users, was not attempted in the previous study. Therefore, the results of the current ICT-based centralized clinical trial can provide new perspectives in PHR-based clinical trials for patients requiring self-adjustment of insulin dose.

## Additional files

Additional file 1: Informed consent form. (PDF 372 kb)

Additional file 2: ICT-based Centralized Clinical Trial Monitoring Questionnaire. (PDF 210 kb)

Additional file 3: Algorithm-based feedback messages for ICT-based intervention group. (PDF 323 kb)

## Abbreviations

App: Mobile application
CRA: Clinical research assistant
CRC: Clinical research coordinator
CTMS: Clinical trial management system
DM: Diabetes mellitus
DTSQ: Diabetes Treatment Satisfaction Questionnaire
eCRF: Electronic case report form
ICT: Information and communication technology
IWRS: Interactive Web Response System
PHR: Personal health record
T1DM: Type 1 diabetes mellitus
T2DM: Type 2 diabetes mellitus
