# Erratum to: An information and communication technology-based centralized clinical trial to determine the efficacy and safety of insulin dose adjustment education based on a smartphone personal health record application: a randomized controlled trial

**DOI:** 10.1186/s12911-017-0534-1

**Published:** 2017-12-12

**Authors:** Gyuri Kim, Ji Cheol Bae, Byoung Kee Yi, Kyu Yeon Hur, Dong Kyung Chang, Moon-Kyu Lee, Jae Hyeon Kim, Sang-Man Jin

**Affiliations:** 1 Division of Endocrinology and Metabolism, Department of Medicine, Samsung Medical Center, Sungkyunkwan University School of Medicine, Seoul, 135-710 Republic of Korea; 2 Division of Endocrinology and Metabolism, Department of Medicine, Samsung Changwon Hospital, Sungkyunkwan University School of Medicine, Changwon, Republic of Korea; 3 Department of Digital Health, SAIHST, Sungkyunkwan University, Seoul, Republic of Korea; 4 Division of Gastroenterology, Department of Medicine, Samsung Medical Center, Sungkyunkwan University School of Medicine, Seoul, Republic of Korea; 5 Department of Clinical Research Design & Evaluation, SAIHST, Sungkyunkwan University, Seoul, Republic of Korea

## Erratum

After publication of the original article [1] it was noted that both the figures and captions and relating to Figs. 1 and 2 had been interchanged.

**Fig. 1:**
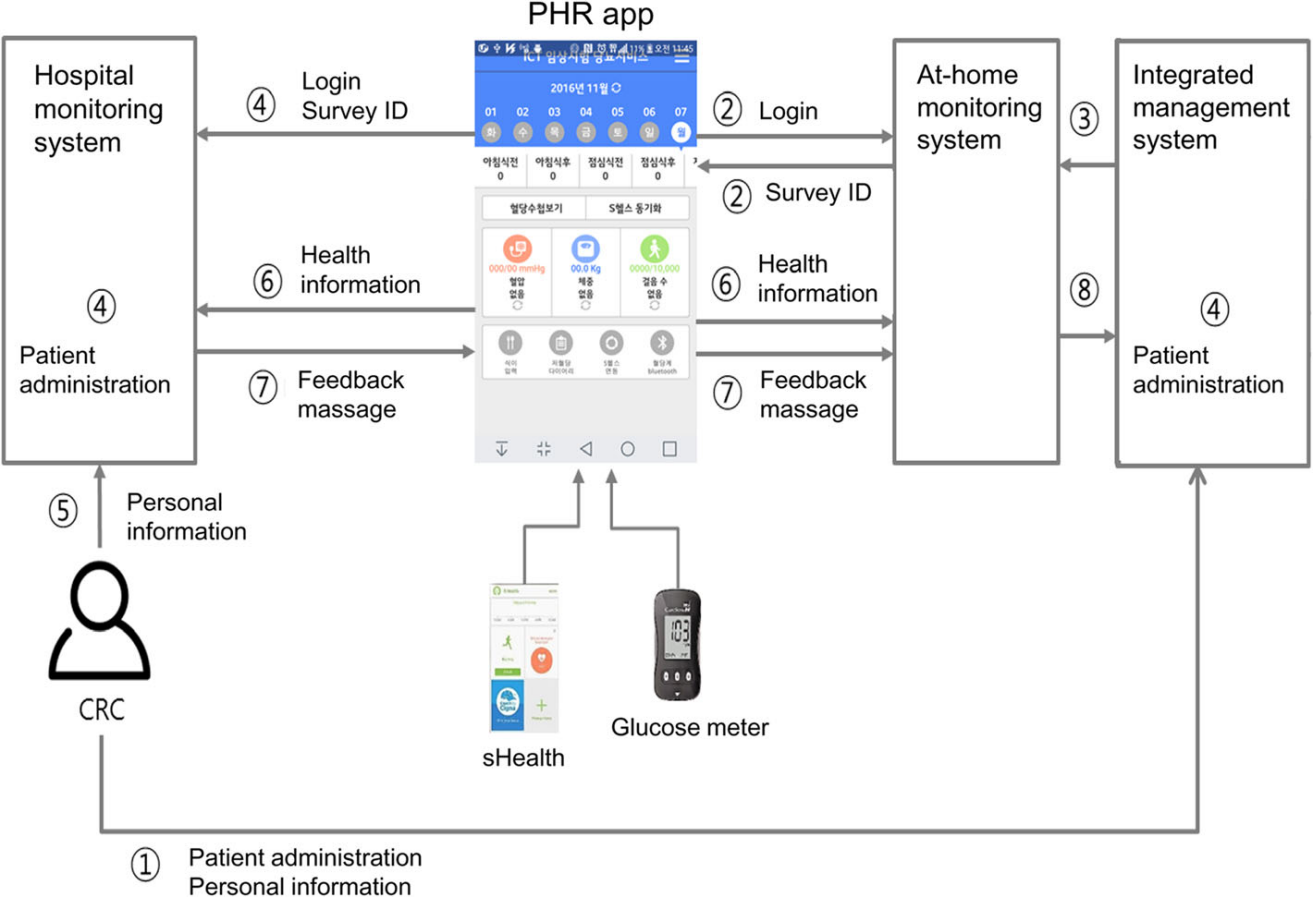
Information and communication technology-based centralized monitoring system. CRC: Clinical research coordinator; PHR: Personal health record. The CRC registers the subject in the integrated management system, registers personalized conditions such as target blood glucose, and logs in the personal health record app through the newly created user ID and password. The PHR app sends login information to the home monitoring system and returns the survey ID. The home monitoring system receives user information (personal information, screening number, etc.) from the integrated management system. The PHR app sends login information and survey ID to the hospital monitoring system and automatically registers the patient. The CRC registers personalized conditions, such as target blood glucose and subject classification, in the hospital monitoring system. Blood glucose values by glucometer, step count by Samsung Health app, insulin regimen, hypoglycemia diary, and food diary inputted by the patient are transmitted to the home and hospital monitoring systems through the HL7 v2 message. Based on the received blood glucose values, the hospital monitoring system sends an algorithm-based feedback message, and the PHR app shows the feedback message. The PHR app that receives the feedback message transmits the HL7 v2 message to the home monitoring system. All information and feedback messages saved in the home monitoring system are stored in the eCRF

**Fig. 2:**
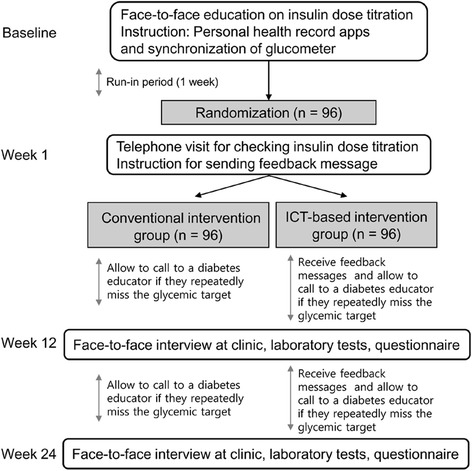
Study flow chart

These errors were introduced during typesetting; thus the publisher apologizes for this error. The correct figures and corresponding captions are shown below.
