# Supplementary material for: An information and communication technology-based centralized clinical trial to determine the efficacy and safety of insulin dose adjustment education based on a smartphone personal health record application: a randomized controlled trial
Source: BMC Med Inform Decis Mak. 2017 Jul 18;17:109. doi: 10.1186/s12911-017-0507-4 (PMC5516303; doi:10.1186/s12911-017-0507-4)
Supplement: Supplementary file 1 — Informed consent form. (PDF 372 kb) [file 12911_2017_507_MOESM1_ESM.pdf]

Consent

Consent version and date	Version 2.0 Date: April. 05. 2017
**Title of Study**	**An information and communication technology-based centralized clinical trial to determine the efficacy and safety of insulin dose adjustment education based on a smartphone personal health record application: a randomized controlled trial**

1. Participation

Investigator of this clinical trial will receive your consent to participate in the clinical trial in accordance with the relevant rules, legal process from ethical principles based on the Helsinki Declaration. You should read this consent carefully before deciding whether or not you will participate in the study. It is important that you understand why this clinical trial is carried out and what it does. You can ask all the questions about this clinical trial and have enough time to decide to participate in this clinical trial. If you received answers of all the questions you have asked and decided to take part in this clinical trial, please sign and date this consent form.

2. This clinical trial is conducted for research purposes

This clinical trial validates the safety and efficacy of insulin dose regimen training based on at-home measurement devices and smartphone personal health record application in patients with diabetes who initiate or intensify their insulin regimen. In addition, we will investigate the stability and validity of ICT-based centralized clinical trial monitoring system by conducting two analyzes using ICT-based or questionnaire/medical record-based evaluation variables.

3. Introduction and purpose of the clinical trial

Patients with diabetes who initiate insulin or intensify their insulin regimen require intensive monitoring of blood glucose levels. Unpredictable hypoglycemia can occur during sleep or driving, and unexpected changes in blood glucose level can put someone else or yourself at risk. Therefore, if you are diagnosed with diabetes, you will record a Diabetes Diary for systematic diabetes management. The Diabetes Diary is essential to understand premeal and postprandial blood glucose levels as well as the effects of lifestyle modification including dietary and exercise at a glance. If management of diabetes is not done properly, it will increase the risk of complications, and sometimes life threatening, so the strict glucose control is important. However, it is often troublesome or forgetful to record everyday, and there is a discomfort that you have to carry your Diabetes Diary. Recently, smart blood glucometers have been released and these devices have simple functions such as automatic saving of measured results or displaying the average value. In addition, we want to integrate a system for clinical trial that can store and retrieve personal medical information and biometric information transmitted from at-home measurement device outside of the hospital, based on information and communication technology (ICT) system. This study systemized the blood glucose level obtained from the smart blood glucometer to be transmitted to the medical staff in real time through the individual smartphone. Medical staff can follow hypoglycemic or hyperglycemic events and send algorithm-based feedback messages to the patients that can help glucose control based on Information and Communication Technology (ICT) system. Therefore, we aim to evaluate the efficacy and safety of the ICT system in this clinical trial.

Patients with diabetes who initiate insulin or intensify their insulin regimen require intensive monitoring of blood glucose levels. Unpredictable hypoglycemia can occur during sleep or driving, and unexpected changes in blood glucose level can put someone else or yourself at risk. Therefore, if you are diagnosed with diabetes, you will record a Diabetes Diary for systematic diabetes management. The Diabetes Diary is essential to understand premeal and postprandial blood glucose levels as well as the effects of lifestyle modification including dietary and exercise at a glance. If management of diabetes is not done properly, it will increase the risk of complications, and sometimes life threatening, so the strict glucose control is important. However, it is often troublesome or forgetful to record every day, and there is a discomfort that you have to carry your Diabetes Diary. Recently, smart blood glucometers have been

released and these devices have simple functions such as automatic saving of measured results or displaying the average value. In addition, we want to integrate a system for clinical trial that can store and retrieve personal medical information and biometric information transmitted from at-home measurement device outside of the hospital, based on information and communication technology (ICT) system. This study systemized the blood glucose level obtained from the smart blood glucometer to be transmitted to the medical staff in real time through the individual smartphone. Medical staff can follow hypoglycemic or hyperglycemic events and send algorithm-based feedback messages to the patients that can help glucose control based on Information and Communication Technology (ICT) system. Therefore, we aim to evaluate the efficacy and safety of the ICT system in this clinical trial.

4. Information of medical device, participant selection and randomization

The clinical trial study consists of 1) at-home measurement device (Bluetooth-enabled blood glucometer including paper strips and lancet) which acquires your health biometric information, and 2) a personal health record application that receives and saves biometric information from at-home device and transmits the information to the server. You will be provided the at-home measurement device and download the personal health record application. Synchronization of the data between the at-home measurement device and the application will be checked and instruction of the application will be guided. Measurement of glucose levels a total of 7 or more times a day including daily at premeal 3 times, post-meal 3 times, and bedtime is recommended. The levels of blood glucose on Bluetooth-enabled blood glucometer will be transmitted to the personal health record application automatically when the Bluetooth is turned on. The step count taken per day acquired by the Samsung Health application (Samsung Electronics, Korea) is automatically linked to the personal health record application. Other information including food diary and insulin dose will be directly recorded on the personal health record application by subject. Subjects will be randomly assigned to either the ICT-based intervention group or the conventional intervention group at a ratio of 1:1 which is open-label, and the randomization is stratified according to the use of pre-prandial rapid-acting insulin two or more times per day. If the subject in ICT-based intervention group has hypoglycemia or hyperglycemia

recorded on the personal health record application, algorithm-based feedback message will be sent through the application. You must follow the guidelines from feedback message. Whether you check your application, or whether you follow the guidelines (re-administration of blood glucose level, counseling phone call) will be checked on the medical staff webpage. The diabetic educator will call you if it is non-compliant and unsynchronized. Subjects in the conventional intervention group also have the stored like the ICT-based intervention group, but algorithm-based feedback messages are not sent. Subjects in the conventional intervention group will manage their glucose levels according to the goal of blood glucose control and instruction. This is no different from the current medical standard. This can be an important resource for deciding the treatment plan because it has the advantage of being a basis for consultation with medical staff when they visit outpatient clinic. In both the conventional and ICT-based intervention groups, you can call the diabetes educator at any time and get counseling if you have any questions about your blood glucose control.

5. Protocol

The following are the steps you will take to participate in this trial from enrolment to completion. You will visit your doctor in outpatient clinic and your doctor will explain the purpose of the study. If you decide to participate in this study, you will be asked to read and sign this consent. After this consent, physical examinations, check for vital signs, laboratory tests, and body composition examination will be performed. When the investigator has determined all of your test results and determines whether they are suitable for clinical trials, you will take part in the clinical trial, according to following protocol. All subjects in the control and intervention groups will be contacted by the clinical research coordinator at the start of the study and will be provided a Bluetooth-enabled glucometer and instructions for the usage and error handling of the personal health record application.

(1) Distribution of an at-home measurement equipment (a Bluetooth-enabled glucometer), download of the personal health record application, and instruction

(2) Education on insulin injection, insulin dose adjustment, and prevention for hypoglycemia by

diabetes educator and provided a guidebook.

(3) Measurement of glucose with Bluetooth-enabled glucometer and synchronization of glucose levels

① Check glucose levels by glucometer for a total of 7 or more times a day (every premeal 3 times a day, postprandial 3 times a day, before going to bed once a day)

② Keep the Bluetooth of the mobile phone turned on to automatically synchronize the measured values from the glucometer to be transmitted and stored.

③ Subjects must examine level of blood glucose through a Bluetooth-enabled glucometer for a 1-week run-in period and synchronize the measured glucose levels to the personal health record application at least once a day during that week. If synchronization is unsuccessful, the subject will be considered as a screening failure and should return the at-home measurement equipment.

(4) Algorithm-based feedback message transmission through medical staff web page (ICT-based conventional group only)

① Your blood glucose levels and health information will be sent to the medical staff web page through the personal health record application. Based on the information transmitted, you will receive feedback messages for blood glucose management. You must check and follow the feedback messages.

② The list of the subjects whose algorithm-based feedback messages are sent through the personal health record app is reported to the diabetes educator through the medical staff web pages and whether the subjects check the feedback messages is also reported. The diabetes educator may check this and send you an additional algorithm-based feedback message or call you.

(5) Laboratory test is performed at visit 1 for screening, visit 2, visit 3 for screening, and fasting for 8 hours is necessary. For women of childbearing age, urine pregnancy test is performed.

(6) Visits 2 and 3 require the diary to be recorded for three days before the visit through the

personal health record application

(7) Satisfaction evaluation by Diabetes Treatment Satisfaction Questionnaire (DTSQ) and a questionnaire for ICT-based centralized clinical trial monitoring will be performed.

(8) Concomitant medications and adverse event assessments will be performed and the clinical trial will be finished

[Study schedules]

	STUDY PERIOD				
	**Screening**	**Run-in**	**Allocation**	**Post-allocation**	
**Visit type**	Visit 1<br>Clinic		Visit 2<br>Tele	Visit 3<br>Clinic	Visit 4<br>Clinic
**Time point**	-4~0 week	-1~0 week	0 week	12 week	24 week
**Visit window (day)**	0		±2	±7	±7
**ENROLMENT**					
Informed consent	X				
Demographic information (sex, age, alcohol consumption, smoking history)	X				
Medical history and drug information	X				
Inclusion/exclusion criteria	X		X		
Instruction on and distribution of at-home measurement device	X				
PHR application; download and instruction	X				

Education on insulin injection, insulin dose adjustment, and prevention for hypoglycemia by diabetes educator		X		X[4)]		
Randomization/Allocation				X		
**INTERVENTIONS**						
ICT-based intervention including feedback messages on the PHR application					⟷	
**ASSESSMENTS**						
Anthropometric measurement (height, weight, BMI)		X			X	X
Vital sign		X			X	X
Body composition		X			X	X
Laboratory test		X			X	X
At-home	Measurement of glucose with bluetooth-enabled glucometer		Everyday		1-4 times per day ⟷	
	Synchronization of glucose levels and step count data into PHR application		Everyday		Everyday ⟷	
	Hypoglycemia diary on PHR application (using paper note if an input through the PHR application was unsuccessful)		If occurred		If occurred ⟷	

	3-day food diary on PHR application				X	X
Satisfaction questionnaire		X			X	X

6. Responsibilities for this clinical trial

During the trial period, you must measure your glucose levels by at-home measurement device and synchronize to the personal health record application to transmit the information. In addition, exercise and diet should be carried out as instructed to manage glycemic control. All subjects should record the symptoms of hypoglycemia, the occurrence of night sweats, and the need for someone to help recover from hypoglycemia in their "Hypoglycemia Diary" in the personal health record application. The ICT-intervention group should check and follow the algorithm-based feedback message sent through the personal health record application when hypoglycemia occurs. However, you should be aware that the feedback message may not be available at every moment, and you must respond in accordance with the prior education and printed instructions. The control intervention group does not provide additional feedback messages when the blood glucose level is not within the target, but the recording of the hypoglycemia diary and the synchronization of blood glucose levels should be performed in the same way as the ICT-based intervention group. You can contact the diabetes education office whenever you need.

7. Unverified experimental aspect of the clinical trial

At-home measurement device, the blood glucometer used in this clinical trial is a product approved by the Food and Drug Administration. The personal health record application is a customized management service program developed by Samsung Seoul Hospital for the management of patients with diabetes. Insulin dose titration and other examination for diabetes management are the same as standard care. However, we have not yet proven the superiority of insulin injection training using either general or the personal health record application-based insulin injection training used in this clinical trial, so we will

clarify this through clinical trial.

8. Risk (side effects) or discomfort predicted by participation in this clinical trial

For this study, you may have to check blood glucose levels one to seven times every day and synchronize to store them in your smartphone application. Insulin injections, diets, and exercises can be performed as you are already trained to treat diabetes. Please contact your research coordinator immediately if you have any errors, transmission failures, or malfunctions in your blood glucometer or personal health record application.

9. Expected benefits from participating in this clinical trial

The benefits you expect from participating in this clinical trial are: 1) diabetes education and a personal health record application are provided free of charge. 2) Both conventional and ICT-based intervention groups can control blood glucose levels effectively by using this personal health record application to check blood glucose information at a glance. 3) ICT-based intervention group can be provided algorithm-based feedback messages and guidelines based on the recorded blood glucose levels and control their blood glucose levels.

10. Other treatment options that can be selected for this disease and the potential risk and benefit of the treatment

This study does not evaluate the efficacy of any particular medication or treatment, and will follow the insulin treatment principles of the usual patients with diabetes, so there is no additional risk other than the usual insulin treatment risks. However, general side effects of conventional insulin therapy can occur such as hypoglycemia and weight gain, and also pain and allergic reactions at the injection site could occur but rare, so you should be aware of the precautions to minimize side effects in your insulin injection training.

11. Estimated duration of study and the approximate total number of subjects participating in the study

This trial is scheduled to recruit 96 people in 11 months from two institutions, and 76 people will participate in this institution. A total of 25 to 29 weeks of participation is expected per subject.

12. Compensation/reimbursement or treatment in the event of a clinical trial-related injury

If during the study period you are injured by this study, you will be compensated in accordance with the clinical trial patient compensation rule, such as receiving appropriate treatment. In case of side effects that are not related to this study, we should closely monitor and treat them. We will also ensure that your personal information and privacy are not infringed by the centralized monitoring based on information and communication technology, and we will take prompt action when a problem arises.

13. Monetary reward and the additional expected outcome by participating in the clinical trial

For a monetary reward for participating in this clinical trial, from the first visit, 50,000 won will be provided for every regular visit. However, transportation expenses may not be paid for visits that are not scheduled. In addition, diabetes education and examination costs related to this clinical trial (blood test, body composition examination) are supported by research funds. There is no additional cost to you for participating in this clinical trial.

14. Limitation of participation in clinical trial

You may be restricted from participation in this study without despite your consent if you fail to follow the directions of your examiner, or if your condition is not tolerable to participate clinical trial.

15. New information that can influence the willingness to continue clinical trial

Participation in this study is entirely your choice. We will notify you or your representative when new information is collected that may affect your willingness to continue participating in this study.

16. Procedures after consent to withdrawal and discontinuation from the clinical trial

Participation in this trial is at your own discretion, and you will not be penalized if you do not participate in the clinical trial. If you want to stop participating in this clinical trial, you can always withdraw your participation, and there will be no disadvantages in continuing to receive treatment at this hospital and you receive the same treatment without discrimination with other patients.

17. Privacy

If you participate in this clinical trial, we will collect your personal information (personally identifiable information such as your name and health information). The collected personal information is strictly controlled in accordance with the relevant laws and regulations, and only research personnel can access the collected data. Of the personal information collected, personally identifiable information is used only for the purpose of linking you with the examination and clinical data obtained through the clinical trial. Your personal information will be used until the study purpose is achieved, and the collected information will be properly managed according to the privacy laws. The record of your identity will be kept confidential even if the results of the clinical trial are published. However, in order to verify the protocol and data quality of clinical trial in accordance with the relevant laws and regulations, the subject's medical records shall be assessed by monitoring associate, inspector, the IRB, or the Food and Drug Safety Department, the confidentiality of the subject's personal information will be protected as much as possible. Access to the data is allowed according to your (or your representative) signed consent form.

18. Provide information on the rights and interests of the subject

This clinical trial was reviewed and approved by the Institutional Review Board (IRB) at Daegu

Clinical Research Ethics Committee, which is responsible for protecting the rights, safety and welfare of the subject. If you have any questions about your rights as a subject of this study, please contact the Institutional Review Board (IRB) at Daegu Clinical Research Ethics Committee (053-623-8490) or Samsung Medical Center (02-3410-2980). If you decide to participate in this clinical trial, you will receive a copy of the signed consent form.

19. Contact

Before signing this form, you must ask all questions that you do not understand. The investigator and clinical research coordinator will answer your questions at any time. If you have any questions about the clinical trial or the progress of this trial, please contact us.

If you have any questions, problems, or adverse events during the study, please contact the investigator, clinical research coordinator, or educator below.

Principal investigator:

Call: ☎ Mobile:

Diabetes educator:

Call: ☎ Mobile:

Clinical research coordinator:

Call: ☎ Mobile:

Clinical Trial Subject Consent Form

- ✓ I have been fully informed of the consent and have consulted with the investigator and research coordinator.
- ✓ I have read, understood and have the opportunity to ask questions and have received satisfactory answers to all my questions.
- ✓ I voluntarily agree to participate in this clinical trial. Even if I sign this agreement, I will not give up my rights.
- ✓ I understand that I can withdraw my consent to this trial at any time and that this will not affect my treatment or my rights.
- ✓ I understand that I will be provided a copy of a signed and dated written statement and agreement after my consent.

Participant's name: ______________________________

Signature: ______________________________ Date: ______________________________

Legal representative: ______________________________ Relationship with participant: ____________

(If needed)

Signature: ______________________________ Date: ______________________________

Investigator's name: ______________________________

Signature: ______________________________ Date: ______________________________

If the subject expresses his or her intention not to be able to read, the investigator read this consent to the subject and discuss this with the subject and confirm the opportunity to ask questions.

Observer's Name: ______________________________ Remarks (relationship, etc.): ____________

(For those who cannot read)

Signature: ____________________________________ Date: ________________________________
