# Supplementary material for: An information and communication technology-based centralized clinical trial to determine the efficacy and safety of insulin dose adjustment education based on a smartphone personal health record application: a randomized controlled trial
Source: BMC Med Inform Decis Mak. 2017 Jul 18;17:109. doi: 10.1186/s12911-017-0507-4 (PMC5516303; doi:10.1186/s12911-017-0507-4)
Supplement: Supplementary file 2 — ICT-based Centralized Clinical Trial Monitoring Questionnaire. (PDF 210 kb) [file 12911_2017_507_MOESM2_ESM.pdf]

## ICT-based Centralized Clinical Trial Monitoring Questionnaire

1. Have you experienced any symptoms of hypoglycemia during the past 3 months (e.g. cold sweating, hunger, shivering, or panic)?

1) Yes 2) No

2. If you answered "1) Yes" in question 1, are you satisfied with the recording way on the hypoglycemia diary on your smartphone regarding the symptoms of hypoglycemia, whether it occurs during night or sleep, and whether you need help to recover from hypoglycemia?

| Satisfied with recording on a smartphone app | 3 | 2 | 1 | 0 | -1 | -2 | -3 | Satisfied with recording on paper or face-to-face counseling |
|---|---|---|---|---|---|---|---|---|

3. Are you satisfied with the way your blood glucose meter is linked to your smartphone app to record blood glucose?

| Satisfied with recording on a smartphone app | 3 | 2 | 1 | 0 | -1 | -2 | -3 | Satisfied with recording on paper or face-to-face counseling |
|---|---|---|---|---|---|---|---|---|

4. Are you satisfied with the way insulin dose is recorded in the smartphone app?

| Satisfied with recording on a smartphone app | 3 | 2 | 1 | 0 | -1 | -2 | -3 | Satisfied with recording on paper or face-to-face counseling |
|---|---|---|---|---|---|---|---|---|

5. Are you satisfied with the way recording your weight, exercise, and blood pressure on your smartphone app?

| Satisfied with recording on a smartphone app | 3 | 2 | 1 | 0 | -1 | -2 | -3 | Satisfied with recording on paper or face-to-face counseling |
|---|---|---|---|---|---|---|---|---|

6. Throughout the clinical trials, do you think the smartphone app is more convenient than the telephone questionnaire or visit counseling method of recording your information such as blood glucose, insulin dose, and hypoglycemic symptoms?

| Satisfied with recording on a smartphone app | 3 | 2 | 1 | 0 | -1 | -2 | -3 | Satisfied with recording on paper or face-to-face counseling |
|---|---|---|---|---|---|---|---|---|

7. Throughout the clinical trial, do you think the smartphone app is more accurate than the telephone questionnaire or visit counseling method of recording your information such as blood glucose, insulin dose, and hypoglycemic symptoms?

| Satisfied with recording on a smartphone app | 3 | 2 | 1 | 0 | -1 | -2 | -3 | Satisfied with recording on paper or face-to-face counseling |
|---|---|---|---|---|---|---|---|---|
