# Supplementary material for: An information and communication technology-based centralized clinical trial to determine the efficacy and safety of insulin dose adjustment education based on a smartphone personal health record application: a randomized controlled trial
Source: BMC Med Inform Decis Mak. 2017 Jul 18;17:109. doi: 10.1186/s12911-017-0507-4 (PMC5516303; doi:10.1186/s12911-017-0507-4)
Supplement: Supplementary file 3 — Algorithm-based feedback messages for ICT-based intervention group. (PDF 323 kb) [file 12911_2017_507_MOESM3_ESM.pdf]

Algorithm-based feedback messages for ICT-based intervention group

1. Basis for algorithm-based feedback messages

① If measured blood glucose value more than once <70mg/dL, patients receive common feedback messages and record hypoglycemia diary on personal health record (PHR) apps

② If the blood glucose value is not within the target range, patients receive algorithm-based feedback messages via PHR apps, and allow to call a diabetes educator.

2. Algorithm-based feedback message

* If the basal insulin is Tresiba or Tuojeo, basal insulin dose titration is performed no more than every 3-4 days even if blood glucose level is not within the target range, according to the preliminary education contents.

A. When use pre-meal rapid acting insulin

① If hypoglycemia (blood glucose <70mg/dL)

-When your blood glucose level is below 70 mg/dL, you are having hypoglycemia. First, eat three candies or drink a half glass of juice, and then immediately after the meal, inject basal insulin less than the dose used on the previous day. For more information, please contact the diabetes education office.

-Record the cause, symptoms, and treatment of hypoglycemia in the hypoglycemic diary.

② If pre-breakfast blood glucose is lower than the target range (e.g. <80 mg/dL or <100 mg/dL),

-The goal of fasting blood glucose is 100-140 mg/dL (individualized target will be shown). Because fasting blood glucose is lower than the target range, a reduction of basal insulin dose is needed. Check the education contents of basal insulin titration regimen. For more information, please call the Diabetes Education Office (080-970-9700).

-Check if you had enough meat, fish, tofu, etc. at the previous dinner.

③ *If blood glucose is lower than the target range (e.g. <80 mg/dL or <100 mg/dL) before lunch, dinner, or bedtime.*

-When your blood sugar level is still low before lunch, dinner, or bedtime even after having a proper amount of meal, you need to reduce the rapid-acting insulin dose.

-For more information, please call the Diabetes Education Office (080-970-9700).

④ *If fasting blood glucose value in the morning is within the target range (individualized, 100-140 mg/dL or 80-130 mg/dL),*

Your fasting blood glucose level is within the target range (e.g. 100-140 mg/dL). Same dose of basal insulin with the previous day is needed.

⑤ *If fasting blood glucose value in the morning is higher than the target for 2 consecutive days (e.g. >130 or >140 mg/dL),*

-Your target of fasting blood glucose level is 100-140 mg/dL (individualized target). As the fasting blood glucose levels is high, higher dose of basal insulin is needed. Check the education contents for basal insulin titration.

-Check if you had heavy dinner including meat, fish, tofu, nuts, or fatty foods at the previous night. If there was nighttime hypoglycemia, the fasting blood glucose in the morning could be high as a reaction. For more information, please call the Diabetes Education Office (080-970-9700).

⑥ *If fasting blood glucose value in the afternoon, at dinner, or bedtime is >200 mg/dL (once or more; immediately),*

-The heavy amount of prior meal or snack could be increase blood glucose level at next meal. Check a proper amount of meal.

-When fasting blood glucose level is still high even after having a proper amount of meal, titration

for rapid-acting insulin is needed. Check the education contents for rapid-acting insulin titration.

-For more information, please call the Diabetes Education Office (080-970-9700).

⑦ *If postprandial blood glucose value after breakfast, lunch, or dinner is >250mg/dL (once or more; immediately),*

-When you have more foods containing carbohydrates (rice, bread, noodles, sweets, fruits, potatoes, sweet potatoes, etc.), higher dose of rapid-acting insulin is needed.

-When postprandial blood glucose level is still high even after having a proper meal, titration for rapid-acting insulin is needed. Check the instruction for rapid-acting insulin titration. For more information, please call the Diabetes Education Office (080-970-9700).

B. When do not use pre-meal rapid acting insulin

① *If hypoglycemia (blood glucose <70mg/dL)*

-When your blood glucose level is below 70 mg/dL, you are having hypoglycemia. First, eat three candies or drink a half glass of juice, and then immediately after the meal, inject basal insulin less than the dose used on the previous day. For more information, please contact the diabetes education office.

-Record the cause, symptoms, and treatment of hypoglycemia in the hypoglycemic diary.

② *If pre-breakfast blood glucose is lower than the target range (e.g. <80 mg/dL or <100 mg/dL),*

-The goal of fasting blood glucose is 100-140 mg/dL (individualized target will be shown). Because fasting blood glucose is lower than the target range, a reduction of insulin dose is needed. Check the education contents of basal insulin titration regimen. For more information, please call the Diabetes Education Office (080-970-9700).

-Check if you had enough meat, fish, tofu, etc. at the previous dinner.

③ *If fasting blood glucose value in the morning is higher than the target for 2 consecutive days (e.g. >130 or >140 mg/dL),*

-Your target of fasting blood glucose level is 100-140 mg/dL (individualized target). As the fasting blood glucose levels is high, an increase in basal insulin dose with a 10% of those dose is needed. Check the education contents for basal insulin titration.

-Check if you had heavy dinner including meat, fish, tofu, nuts, or fatty foods at the previous night. If there was nighttime hypoglycemia, the fasting blood glucose in the morning could be high as a reaction. In this case, a reduction of basal insulin dose is needed.

For more information, please call the Diabetes Education Office (080-970-9700).
